# Genetic variations in DNA repair gene *NEIL1* associated with radiation pneumonitis risk in lung cancer patients

**DOI:** 10.1002/mgg3.1698

**Published:** 2021-06-09

**Authors:** Yuming Zheng, Leizhen Zheng, Jiahua Yu, Mawei Jiang, Songfang Zhang, Xuwei Cai, Meiling Zhu

**Affiliations:** ^1^ Department of Oncology Xinhua Hospital Affiliated to Shanghai Jiao Tong University School of Medicine Shanghai China; ^2^ Department of Radiation Oncology Shanghai Chest Hospital Affiliated to Shanghai Jiao Tong University School of Medicine Shanghai China; ^3^ Department of Radiation Oncology Xinhua Hospital Affiliated to Shanghai Jiao Tong University School of Medicine Shanghai China

**Keywords:** genetic variations, lung cancer, *NEIL1*, radiation pneumonitis, radiotherapy

## Abstract

**Background:**

Radiation pneumonitis (RP) is a common side effect in lung cancer patients who received radiotherapy. Our previous study found genetic variations in DNA repair gene *NEIL1* may be a predictor of RP in patients with esophageal cancer. So, we hypothesis genetic variations in *NEIL1* gene could affect the risk of RP in lung cancer patients following radiotherapy.

**Methods:**

Genetic variations rs4462560 G>C and rs7402844 C>G in *NEIL1* gene were genotyped in 174 lung cancer patients received radio(chemo)therapy. Luciferase assay, real‐time PCR and Western blot were used to access the effect of the variants on *NEIL1* in HELF and HEF cell lines which were transfected with plasmids containing rs4462560 G>C and rs7402844 C>G.

**Results:**

Patients with rs4462560 CC genotype had a lower risk of RP grade ≥2 than GG genotype. Compared with the CC genotype, rs7402844 GG genotype was associated with an increased RP grade ≥2 risk. What is more, rs4462560 G decreased the relative luciferase activity of *NEIL1* gene promoter compared with the negative control in vitro, while rs4462560 C can increase the relative luciferase activity. The mRNA and protein level of the *NEIL1* gene in rs4462560 G were lower than rs4462560 C.

**Conclusions:**

Genetic variants of *NEIL1* are associated with RP risk through regulation of *NEIL1* expression and serve as independent biomarkers for prediction of RP in patients treated with thoracic radiotherapy.

## Graphical abstract

We found genetic variants of NEIL1 are associated with RP risk in the study. These variants might exert their effect through regulation of NEIL1 expression and serve as independent biomarkers for prediction of RP in patients treated with thoracic radiotherapy.

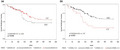

## 1 INTRODUCTION

Radiotherapy plays an important role in curative and palliative treatment for thoracic tumors. Radiation pneumonitis (RP), an acute clinical symptom of radiation‐induced lung injury (RILI), is one of the most common side effect of lung cancer patients after radiotherapy(Giuranno et al., 2019). It not only restricts the clinical efficacy during radiotherapy, but also adversely affects the prognosis of patients. In some cases, RP can be lethal cause on radiotherapy patients, the mortality of severe RP even up to 50% (Wang et al., 2002). Hence, it's pivotal to explore the mist clouding covered on mechanism of RP, searching for valid predictive factors, identifying high‐risk groups of RP patients, and tailoring individualized radiotherapy for different patients.

Lung tissue, especially lower respiratory, is exposed to high oxidative stresses, including oxygen exchange, smoking, ionizing radiation. In lung cancer patients who accept radiotherapy, reactive oxygen species (ROS) and free radicals generate immediately in target area then induce DNA damage after irradiation subsequently. Numerous DNA repair genes mobilize to rescue those damages immediately. The loss of DNA repair capacity results in genetic instability that may lead to a decline of cellular function and play an important role in predisposition to RP.

*Nei endonuclease VIII*‐*like 1* (*NEIL1*) (OMIM*608844), located at chromosome 15q24.2, is an important bifunctional enzyme in initial of base excision repair (BER) pathway, with both glycosylase and lyase activities. Originally, *NEIL1* was uncovered as a substitute for *NTH1* and *OGG1*, since this enzyme could excise 8‐oxoguanine, 5‐hydroxycytosine and basic sites in Nth1(‐/‐) and Ogg1(‐/‐) mice (Morland et al., 2002). Subsequent research has found that *NEIL1* excise a range of oxidatively damaged bases in ssDNA, dsDNA, and bubble DNA, which means this glycosylase possesses a broad substrate specificity. *NEIL1* also interacts with many proteins include *PARP*‐*1*, *XRCC1*, *WRN*, *PCNA*, *RPA* and *RAD9* by its C‐terminal region. The function of *NEIL1* is highly conserved from prokaryote to eucaryon. However, the deletion of *NEIL1* gene in the genome or inhibition its function after translation will interfere with efficient excision of oxidative lesions from chromatin, which may result in instability in the genome and cell apoptosis. Previously research found depletion of *NEIL1* sensitized human embryonic kidney (HEK) 293 cells to ROS significantly (Hegde et al., 2012). Ionizing radiation (IR) significantly increased the DNA damage in Neil1(−/−) mice and exhibit more apoptosis in their hippocampus (Yang et al., 2019). Recently study demonstrated that *NEIL1* intracellular is IR induced in response to, specifically by a Mule‐dependent manner (Edmonds et al., 2017). Besides, *NEIL1* could repair a variety of mutagenic and cytotoxic bases, such as 5,6‐dihydrouracil (DHU), 5‐hydroxycytosine (5‐OHC), 5‐hydroxyuracil (5‐OHU) and spiroiminodihydantoin (Sp) et al, most of which are produced by IR. Thus, we hypothesize *NEIL1* is an important BER gene acts in radiation pneumonitis.

Single nucleotide polymorphism (SNP) is an important genetic variation in human. The relationship between SNPs and risk of various diseases are under research. NIH SNPinfo website (http://snpinfo.niehs.nih.gov/snpfunc.html) and Alibaba2.1 online software (http://www.gene regulation.com/pub/programs/alibaba2/) were used to screen for functional SNPs, respectively. Hereby, we found mutation of *NEIL1* rs4462560 G>C could promote transcription of *NEIL1* gene by preventing the combination of Represso and transcription factor binding site (TFBS) in *NEIL1*. Our previous study found *NEIL1* rs4462560 G>C may serve as a predictor of RP in esophageal cancer patients who received definitive radiotherapy with or without chemotherapy. Patients who had the rs4462560 CC genotype had a statistically significantly lower risk of PR grade ≥2 compared with patients who had the GG genotype (Chen et al., 2013). Later, a result from patients with recurrent depression disorders (rDD) showed that the C/C genotype and allele C of rs4462560 decreased the risk of rDD occurrence, while the G/G genotype and allele G of the same SNP increased the risk (Czarny et al., 2015), which further proved that rs4462560G>C is a functional site. In order to confirm whether the variant is disease causing or not, we further conducted a study to evaluate the association of *NEIL1* SNPs with RP in lung cancer patients. We also verify the association functionally using luciferase assays, real‐time PCR and Western blot. The aim was to determine a customized treatment regimen for each individual and deliver a radiation dose tailored to a patient's normal tissue sensitivity profile rather than to the average radiation tolerance of the whole population.

## 2 MATERIALS AND METHODS

### 2.1 Ethical compliance

This study has been approved by the ethics committee of the Shanghai Chest Hospital, Shanghai, China, we have also acquired the written informed consent from participating patients.

### 2.2 Patients

The study was performed in accordance with the ethical standards of the Ethical Committee of Shanghai Chest Hospital. Patients were recruited between January 2017 and December 2018 at the Department of Radiotherapy, Shanghai Chest Hospital. All patients signed informed consent. The eligible patients were as follows: (1) newly confirmed diagnosis of lung cancer with pathology; (2) received intensity modulated radiotherapy (IMRT) with or without chemotherapy; (3) blood samples were collected before therapy. Lung condition was evaluated weekly during radiotherapy for each patient, and chest X‐ray or chest CT were reviewed at the end of radiotherapy and within 3 months after treatment. Clinical and follow‐up information was abstracted from hospital medical records, while dosimetric data were calculated using the Philips Healthcare radiation therapy planning system (Pinnacle 8.0; Philips Radiation Oncology Systems, Milpitas, Calif) for each patient, including the mean lung dose (MLD), and the volume of lung that received at least 20 Gy (V20). The following potential risk factors were investigated: sex, age (≤65 years versus >65 years), disease stage (8th TNM classification, I‐II versus III‐IV), smoking status (yes versus no), chemotherapy (yes versus no), radiation dose (≤60 Gy vs. >60 Gy), fractioned dose (≤200 mGy vs. >200 mGy), MLD (≤13 Gy vs. >13 Gy), V20 (≤0.2 vs. >0.2). All data collection was completed by two researchers independently and achieved a consensus on each item.

### 2.3 Evaluation of RILI

The time to acute development of RP was calculated from the initial radiotherapy to the final analysis (November 3, 2019). Common Terminology Criteria for Adverse Events (CTCAE) version 5.0 was used to assess the grade of RILI as follows: grade 0, no change; grade 1, asymptomatic, clinical or diagnostic observations only, intervention not indicated; grade 2, symptomatic, medical intervention indicated, limiting instrumental ADL (activities of daily living); grade 3, severe symptoms, limiting selfcare ADL, oxygen indicated; grade 4, life‐threatening respiratory compromise, urgent intervention indicated (e.g., tracheotomy or intubation) and grade 5, death. (https://evs.nci.nih.gov/ftp1/CTCAE/CTCAE_5.0/NCIt_CTCAE_5.0.xlsx). The occurrence and severity of RP were determined within 3 months after radiotherapy. RP grade ≥2 was regarded as the endpoint. Time to event (RP grade ≥2) was based on the duration from start of radiation treatment to occurrence of toxicity.

### 2.4 DNA extraction and genotyping

Genomic DNA was extracted from venous blood using TIANamp Genomic DNA Kit (TIANGEN BIOTECH, Beijing, China) according to the protocols described by the manufacturers and stored at −80°C. The purity and concentrations of the DNA samples were measured spectrophotometrically by calculating ration between absorbance at 260 and 280 nm. Genotypes of the rs4462560 G>C and rs7402844 C>G were done using TaqMan SNP Genotyping Assay according to manufacturer's instruction. Real‐time PCR was performed in a final volume of 10 µl containing 5 µl 2 × TIANexact Genotyping PreMix (Probe), 0.2 µl 50 × ROX Reference Dye, 0.25 µl 20 × SNP genotyping assay, and 1.5 µl DNA and 3.05 µl RNase‐free water. The cycling conditions were used as follows: initial denaturation at 95°C for 10 min, followed by 40 cycles of 95°C for 15 s, and 60°C for 1 min. For all genotypes, the assay success rate was >97%, and the repeated sample's results were 100% concordant.

### 2.5 Cell culture

Human embryonic fibroblast cell line (HEF) and Human embryonic lung fibroblast cell line (HELF) (both passage 5) were purchased from the Shanghai Institute of Cell Biology, Chinese Academy of Sciences, and were cultured in Dulbecco's modified Eagle's medium (DMEM) containing with 10% fetal bovine serum (FBS), 2 mM l‐glutamine, penicillin (100 units/ml), and streptomycin (100 µg/ml), at 37℃ in a humidified atmosphere of 5% CO_2_.

### 2.6 Plasmid construction

Take wild cDNA as a template, designing *NEIL1* primer according to known gene sequence (Gene ID: 79661). The primers used were 5′‐CGACGAGCGGAACGGAG‐3′ and 5′‐TCGGTTAATCCTGACTCCCC‐3′. PCR were used to amplify a 1023 bp DNA sequence, which centered by *NEIL1* gene promoter rs4462560 G (wild‐type, WT) and added the BgIII and HindIII restriction endonuclease action sites at both ends. And rs4462560 C (mutation‐type, MT) was amplified in the same method. Then constructed those two primers into pGK‐Gluc vector and got the pGK‐Gluc‐NEIL1‐WT and pGK‐Gluc‐NEIL1‐MT fluorescent plasmids. Restriction analysis and complete DNA sequencing confirmed the orientation and integrity of each construct's insert.

### 2.7 Transient transfection and luciferase assays

Then HEF and HELF cells (3 × 10^5^ cells) were placed in 24‐well plates and used for transfection after 16 hours when adhering cells grown to 70% confluence. Before transfection, Opti‐MEM (Invitrogen) was substitute for previous 10% FBS‐DMEM, then Lipofectamine 2000 (Thermo Fisher Scientific) was used to transfect pGK‐Gluc‐NEIL1‐WT, pGK‐Gluc‐NEIL1‐MT and pGK‐Gluc control vectors into HEF and HELF cells, respectively. 4 h later, transfection medium was replaced by 10% FBS‐DMEM. Cells were fully lysed after 24 h, firefly luciferase activity was detected by Dual‐Luciferase Reporter Assay System. Then Stop&Glo buffer was added to quench firefly luciferase activity and active renilla luciferase activity. The value of luciferase activity to each hole is equal to the ratio of firefly luciferase activity to renilla luciferase activity. Take the luciferase activity of pGK‐Gluc control vectors as the reference value 1, the transcriptional activity of pGK‐Gluc‐NEIL1‐WT and pGK‐Gluc‐NEIL1‐MT were calculated by comparing with the luciferase activity of pGK‐Gluc control vectors, respectively.

### 2.8 Real‐time quantitative analysis of NEIL1 mRNA

Total RNA was isolated from vector‐transfected cell lines with the TRIzol reagent (Thermo Fisher Scientific) and then converted to cDNA using a reverse transcription kit (TAKARA). The primers used for *NEIL1* were 5′‐TCTGCGGGCAGAGATCCTGTA‐3′ and 5′‐GTCTGGATTCTGCAGCTTGGT‐3′; and the primers used for GAPDH, an internal reference gene, were 5′‐CAGGAGGCATTGCTGATGAT‐3′ and 5′‐GAAGGCTGGGGCTCATTT‐3′. Relative gene expression quantitation for *NEIL1* and for *GAPDH* were carried out using an ABI Prism 7300 sequence detection system (Applied Biosystems, Foster City, CA) in triplicate, based on the SYBR‐Green method. PCR specificity was confirmed by dissociation curve analysis. The expression of individual *NEIL1* measurements was calculated relative to the expression of *GAPDH*.

### 2.9 Western blot

RIPA Lysis Buffer (Beyotime) containing phosphatase and proteinase inhibitors (Beyotime) was used to lysed cells washing by cold PBS three times, and then BCA protein assay kit (Beyotime) was utilized to determine the protein concentration. Cell lysates were mixed with SDS‐PAGE sample loading buffer (Epizyme), followed by boiling for 5 min at 100°C. After that, protein (40 µg/lane) was separated by 10% sodium dodecyl sulfate–polyacrylamide gel electrophoresis (SDS‐PAGE) and transferred onto 0.2 μm polyvinylidene fluoride (PVDF) membranes. After blocked membranes with 5% skim milk for two hours at room temperature, primary rabbit antibodies against NEIL1(ab192517, 1:1000) and GPADH (AF1186, 1:1000) were incubated on a shaker overnight at 4°C. Following Tris‐buffered saline Tween‐20 (TBST, Epizyme) washing, the membranes were then incubated with secondary antibodies against rabbit for two hours at room temperature. At last, protein bands were examined by ECL solution (Thermo Scientific) and the bands density was analyzed by ImageJ software.

### 2.10 The eQTL analysis

GTEx portal was used to assess the correlation between *NEIL1* SNP rs4462560, rs7402844 and *NEIL1* gene mRNA expression levels (http://www.gtexportal.org/home/). The latest version (May. 2016 release V8) includes genotype data from approximately 948 donors and 17382 RNA‐seq samples across 54 tissues and 2 cell lines, with sufficient power to detect eQTL in 54 tissues. The associations of genetic variant and gene expression are investigated in various tissue types.

### 2.11 Statistical analysis

All statistical analyses were performed with SAS software (version 9.1; SAS Institute) and GraphPad Prism8. The associations between RILI and clinical data, radiation dose parameters and SNPs of *NEIL1* were determined using Cox proportional hazards model to estimate the hazard ratio (HR) and 95% confidence interval (CI). Univariate and multivariate analyses were performed, respectively. Furthermore, the multivariate analysis was adjusted by covariates including gender, age, smoking, stage, co‐chemotherapy or not, radiation dose, radiation fractionation, MLD and V20, those factors may cause an influence on RILI that had been confirmed in previous studies. Correlation analysis between genotypes and RILI was performed by log‐rank method to test whether the differences between the two groups were statistically significant. Student's *t* test or one‐way analysis of variance (ANOVA) were used to analyze the difference between groups. All analyses adopted standardized two‐tailed test, *p* < 0.05 was defined as the criterion of statistical significance.

## 3 RESULTS

### 3.1 Patients characteristic

The patient characteristics are listed in Table 1. We recruited 183 lung cancer patients in Shanghai Chest Hospital between January 2017 and 2018. Finally, 174 patients completed follow‐up information and provided whole blood samples, of whom 142 (81.6%) were men and 32 (18.4%) were women with a median age of 62 years (range, 38–82 years). Among all patients, 65 (36.4%) were classified as squamous cell carcinoma, 48 (34.1%) adenocarcinoma, 54 (23.1%) small cell carcinoma, and (7 or 4%) others. 30 (17.2%) of patients had stage I/II diseases while 144 (82.8%)had stage III/IV according to the 8th TNM classification. What's more, 117 (67.2%) patients had a history of smoking and 114 (65.5%) treated with a combination of chemo‐radiotherapy. The median radiation dose was 60 Gy (range, 30–63 Gy) with a median MLD of 11.27 Gy (range, 0.97–15.98 Gy). The median occurrence time for acute RP was 45 days (range, 10–365 days). There were occurrences 63 (36.2%) patients suffered RP grade ≥2 and 19 (10.9%) grades ≥3 in the study (The number of patients with RP grade 0, 1, 2, 3 and 4 were observed in 55, 56, 44, 19 and 0, respectively).

**TABLE 1:** Demographic and baseline clinical characteristics of patients

Variable	No.	%
Sex		
Male	142	81.6
Female	32	18.4
Age, years		
Median	62	
Range	38–82	
Histopathology		
Squamous cell carcinoma	65	37.4
Adenocarcinoma	48	27.6
Small cell lung cancer	54	31
Others	7	4
Disease stage		
I	11	6.3
II	19	10.9
III	124	71.3
IV	20	11.5
Smoking status		
Smoker	117	67.2
Nonsmoker	57	32.8
Chemotherapy		
No	60	34.5
Yes	114	65.5
Radiation dose		
Median	60	
Range	30–63	
Fractioned dose		
Median	200	
Range	180–1250	
MLD		
Median	11.27	
Range	0.97–15.98	
V20, %		
Median	20	
Range	0–28	

### 3.2 *NEIL1* SNPs and the risk of acute RP

**FIGURE 1:**
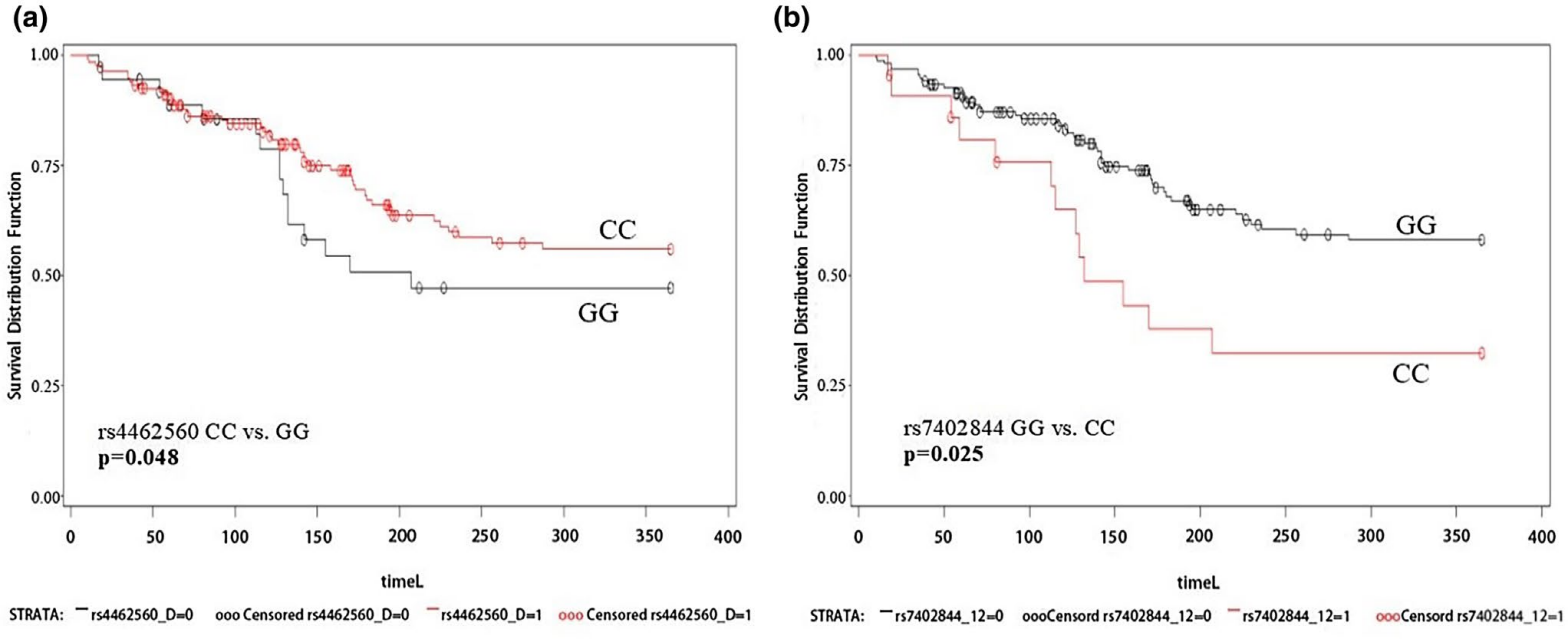
Cumulative probability of Grade ≥2 radiation pneumonitis in 173 patients with non–small cell lung cancer as a function of time from the start of radiation therapy by genotypes: (a) NEIL1 rs4462560 CC vs. GG (b) NEIL1 rs7402844 GG vs. CC

Among all dosimetric parameters, only V20 was associated with RP. V20 >20% was significantly associated with increased risk of grade ≥2 RP according to both univariate and multivariate analysis. (adjusted HR = 1.756, 95% CI = 1.160–2.761, *p* = 0.026) (Table 2). After adjusting for multiple factors, patients with *NEIL1* rs4462560 CC genotype had a reduced risk of acute RP compared with patients with GG genotype (HR = 0.489, 95% CI = 0.240–0.996, *p* = 0.048). In addition, patients with rs7402844 CC genotype had a higher risk of grade ≥2 RP than ones with GG genotype (adjusted HR = 2.338, 95% CI = 1.112–4.914, *p* = 0.025) (Table 3). The log‐rank test found that the curve difference between the two groups was statistically significant (Figure 1).

**TABLE 2:** Clinical and dosimetric parameters and their association with severe RP (grade ≥2) in NSCLC patients who received definitive radiotherapy

Parameter	Univariate analysis		Multivariate analysis	
	HR (95% CI)	*p*	HR (95% CI)	*p* ^a^
Sex				
Male	1.00		1.00	
Female	1.322 (0.703–2.488)	0.386	2.074 (0.775–5.550)	0.147
Age (y)				
≤65	1.00		1.00	
>65	0.659 (0.363–1.196)	0.170	0.753 (0.379–1.496)	0.418
Smoking status^b^				
Nonsmoker	1.00		1.00	
Smoker	1.092 (0.637–1.870)	0.749	1.417 (0.615–3.268)	0.413
Disease stage				
I‐II	1.00		1.00	
III‐IV	1.561 (0.766–3.181)	0.220	1.257 (0.576–2.743)	0.566
Chemotherapy				
No	1.00		1.00	
Yes	1.510 (0.865–2.636)	0.147	1.274 (0.671–2.417)	0.147
Radiation dose^c^				
≤60.0	1.00		1.00	
>60.0	0.548 (0.261–1.150)	0.112	0.449 (0.158–1.280)	0.134
Fractioned dose^d^				
≤200	1.00		1.00	
>200	0.749 (0.448–1.252)	0.270	1.038 (0.498–2.161)	0.921
MLD^c^				
≤13	1.00		1.00	
>13	1.337 (0.773–2.311)	0.298	0.790 (0.391–1.598)	0.512
V20, %				
≤0.20	1.00		1.00	
>0.20	1.934 (1.156–3.235)	0.012	1.756 (1.160–2.761)	0.026

Abbreviations: CI, confidence interval; HR, hazard ratio; MLD, mean lung dose; NSCLC, non‐small cell lung cancer; RP, radiation pneumonitis; V20, the volume of lung that received at least 20 Gy.

^a^ *p* values were calculated by Cox proportional model using univariate analysis with adjustment for sex, age, smoking history, disease stage, chemotherapy history, radiation dose, fractioned dose, MLD and V20.

^b^ Unit year × pack.

^c^ Unit Gy.

^d^ Unit mGy.

**TABLE 3:** Associations between NEIL1 genotypes and RP grade ≥2 in lung cancer patients who received radiotherapy

Genotypes	Patient No.	Event (%)	Univariate analysis		Multivariate analysis	
			HR (95% CI)	*p*	HR (95% CI)	*p* ^a^
rs4462560						
GG	37	16(43.2)	1.00		1.00	
GC	82	29(35.4)	0.810 (0.445–1.475)	0.492	0.628 (0.329–1.197)	0.157
CC	51	17(33.3)	0.708 (0.361–1.387)	0.314	**0.489 (0.240–0.996)**	**0.048**
GC/CC	133	46(34.6)	0.717 (0.406–1.266)	0.251	0.573 (0.312–1.053)	0.073
rs7402844						
GG	73	23(31.5)	1.00		1.00	
GC	75	26(34.7)	1.146 (0.658–1.996)	0.631	1.176 (0.653–2.118)	0.588
CC	22	**13(59.1)**	**2.376 (1.209–4.671)**	**0.012**	**2.338 (1.112–4.914)**	**0.025**
GC/CC	97	39(40.2)	1.360 (0.812–2.278)	0.242	1.420 (0.820–2.458)	0.210

Abbreviations: CI, confidence interval; HR, hazard ratio; NSCLC, non‐small cell lung cancer; RP, radiation pneumonitis.

The bold figures indicates that the *p* value is statistically significant.

^a^ *p* values were calculated by Cox proportional model using univariate analysis with adjustment for sex, age, smoking history, disease stage, chemotherapy history, radiation dose, fractioned dose, MLD and V20.

### 3.3 Allelic gene variation regulating the transcription and translation of *NEIL1* gene

**FIGURE 2:**
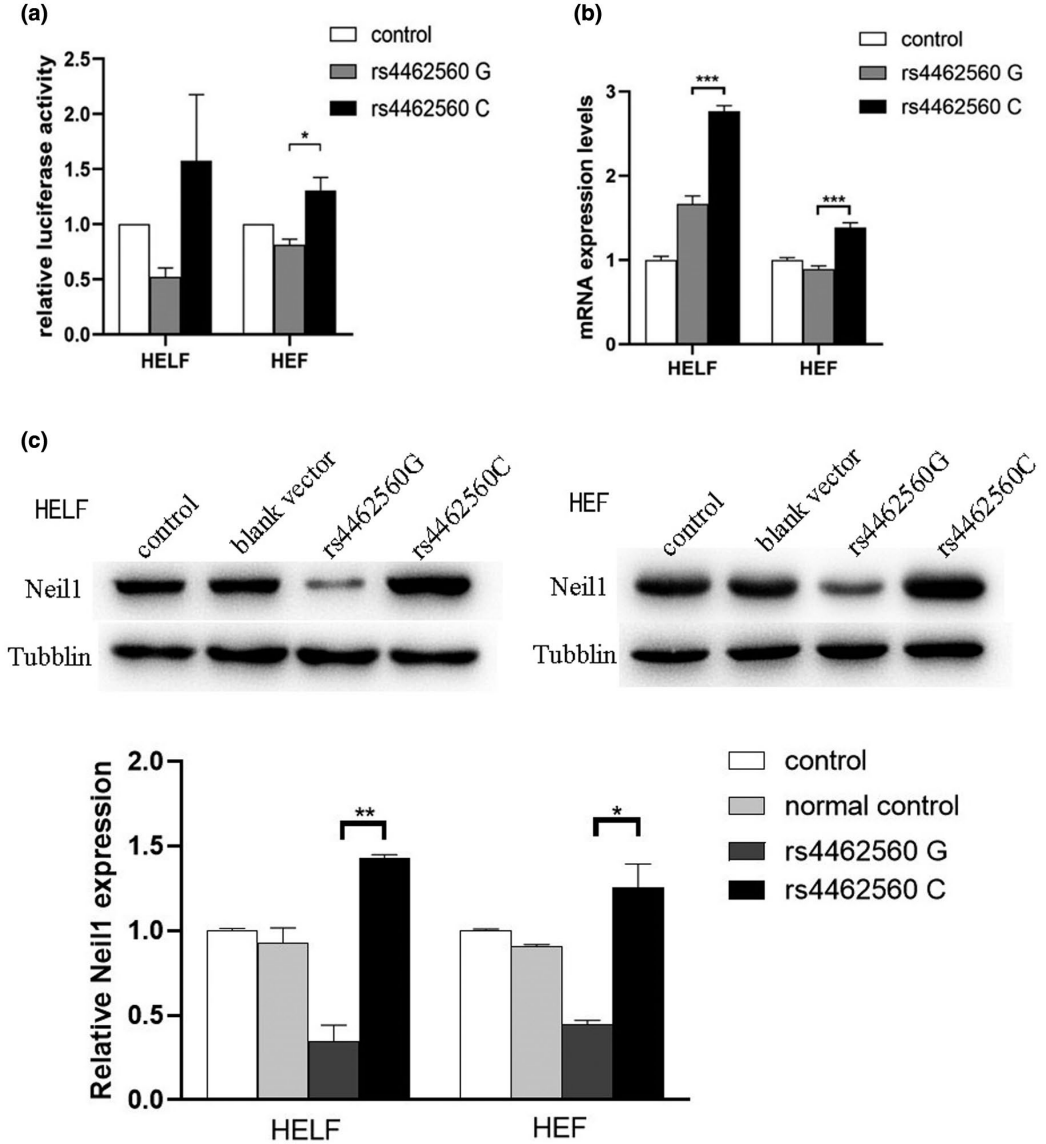
(a) Luciferase assays were used to confirm the role of SNP as a regulation factor of NEIL1 promoter. NEIL1 rs4462560 G decreased activity of NEIL1 gene promoter, and the mutation of rs4462560 C increased activity of NEIL1 gene promoter, those results were observed in both HEF and HELF cells. (b) Q‐PCR were used to detect the influence of rs4462560 G/C on NEIL1 transcription. In HEF cells, SNP rs4462560 G downregulated the expression of NEIL1 mRNA while rs4462560 C upregulated the expression of NEIL1 mRNA. (c) Western blot was used to test the influence of rs4462560 G/C on NEIL1 translation. NEIL1 rs4462560 G decreased the expression of NEIL1 in protein level, while the mutation of rs4462560 C increased the expression of NEIL1, those results were observed in both HEF and HELF. *p < 0.05 compared with each of the constructs. All experiments were performed in triplicate at least in three independent transfection experiments, and each value represents the mean standard deviation. *p < 0.05 compared with each of the constructs. **0.05 < *p* < 0.001 compared with each of the constructs. ***p < 0.001 compared with each of the constructs

**FIGURE 3:**
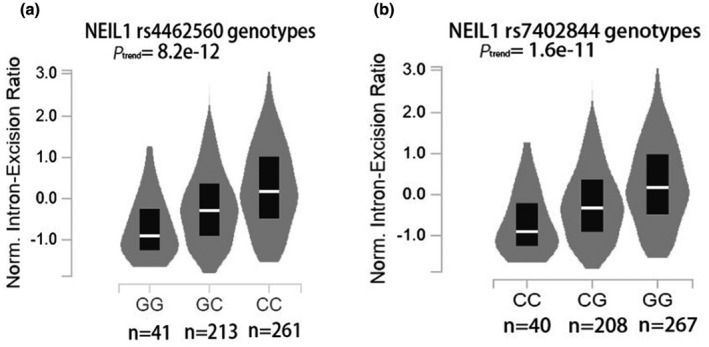
GTEx were used to identify different genotypes affected the expression of NEIL1 mRNA in normal lung tissue adjacent to the tumors. In SNP rs4462560, allelic C upregulated the level of NEIL1 mRNA in lung tissues than allelic genes G. In SNP rs7402844, allelic G upregulated the level of NEIL1 mRNA in lung tissues than allelic genes C (*p*_trend_ = 8.2e‐12 for rs4462560 and *p*_trend_ = 1.6e‐11 for rs7482844)

The effect of different alleles of rs4462560 G> C on the transcription activity of *NEIL1* gene was analyzed by luciferase activity assay, and it found that rs4462560 G decreased the activity of *NEIL1* gene promoter compare with the negative control, while rs4462560 C can increase the activity of *NEIL1* gene promoter in both HEF and HELF (*p* = 0.132 in HELF, *p* = 0.032 in HEF) (Figure 2a); Real‐time PCR analysis of the effects of different alleles of rs4462560 G>C on the transcription level of *NEIL1* gene found that rs4462560 C could upregulate the expression level of *NEIL1* mRNA in normal cell lines than rs4462560 G in both HEF and HELF (*p* = 0.000082 in HELF, *p* = 0.000278 in HEF) (Figure 2b); Western Blot analysis further proved this result that rs4462560 C could upregulate the protein level of *NEIL1* than rs4462560 G in both HEF and HELF(*p* = 0.004 in HELF, *p* = 0.014 in HEF) (Figure 2c). We also searched from the Genotype‐Tissue Expression (GTEx) project aims to illuminate *NEIL1* gene variation in 515 lung tissues of the human body, the conclusion is that allele C in rs4462560 upregulated the expression level of *NEIL1* mRNA in lung tissues (*p*_trend_ = 8.2e‐12, Normal effect size = 0.46, Figure 3a), while allele G in rs7402844 upregulated the expression level of *NEIL1* mRNA in lung tissues (*p*_trend_ = 1.6e‐11, Normal effect size = 0.46, Figure 3b).

## 4 DISCUSSION

Herein, we examined two *NEIL1* genetic variations in 174 lung cancer patients received radiotherapy with or without chemotherapy and investigated their association with the risk of RP. Moreover, we verified one of SNPs in two normal cell lines by functional luciferase assay, real‐time PCR and Western blot. *NEIL1* rs4462560 is localized in the gene promoter of *NEIL1* near 3′ UTR region, which is a missense mutation with a G>C substitution. Another SNP is rs7402844 which is a transition of C>G in the 5′ end of *NEIL1*.

We found a reduced risk of acute RP in rs4462560 CC genotype patients rather than rs4462560 GG carriers after adjusting for multiple factors. In addition, patients with rs7402844 CC genotype had a higher risk of grade ≥2 RP than ones with GG genotype. Luciferase assays proved that rs4462560 C can increase the activity of *NEIL1* gene promoter in both HEF and HELF. Later real‐time PCR results revealed that rs4462560 C could upregulate the expression level of *NEIL1* mRNA than rs4462560 G in two cell lines, which further corroborated previously consequences. Besides, we did not find any association between dosimetric parameters and RP risk except V20, it was according with previous researchers(Zhang et al., 2014). Our results also demonstrated that V20 >20% was significantly predict the risk of grade ≥2 RP. That evidence confirmed V20 was significant risk predictive factors for severe RP, which reaches a consensus with other studies (Grambozov et al., 2020; Tang et al., 2020).

The relationship between *NEIL1* and cancers pathogenesis have been investigated. Data analysis from TCGA database suggested that nine cancer types exhibit epigenetic silencing of the *NEIL1* gene through promoter hypermethylation. Meanwhile the *NEIL1* protein expression level was significantly lower in the tumor tissues than in the nontumor tissues (Shinmura et al., 2016). The latest research on human colorectal cancer (CRC) shows that silence of *NEIL1* in two CRC (HCT116 and SW480) cell lines promoted cell apoptosis through the caspase‐9 signaling pathway. On the contrary, overexpression of *NEIL1* in two cell lines got opposite phenomenon (Xue et al., 2020). All of those results indicated the importance of *NEIL1* in BER pathway, however, we noticed that complications during cancer radiotherapy are also deserved our concern.

It has been suggested in previously studies that the C/C and allele C genotype of *NEIL1* rs4462560 were negatively correlated with recurrent depression disorder (rDD) while genotype G/G and allele G of the same SNP were positively correlated with the disease (Czarny et al., 2015). Chen et al used to find *NEIL1* rs4462560 GC/CC genotype significantly decreased the risk of grade ≥2 acute RP in ESCC patients than GG genotype statistically (Chen et al., 2013). Those results may indicate that the G variant may affect the functionality of *NEIL1*. On the contrary, Zhai et al reported that neither the genotypes nor haplotypes of *NEIL1* rs4462560 were associated with risk of squamous cell carcinomas of the oral cavity and oropharynx (SCCOOP) (Zhai et al., 2008). In Wojcik's study, researchers did not find any correlation between genotypes or alleles of the *NEIL1* rs4462560 and Keratoconus occurrence, however, some associations were observed while combined *NEIL1* SNP with gene variants of *PARP*‐*1*, *POLG*, *XRCC1* (Wojcik et al., 2014). Similar results were obtained in studies on Alzheimer's Disease (AD), data for *NEIL1* rs4462560 showed no significant association with AD risk, but the combination of *NEIL1* rs4462560 and *OGG1* rs1052133 polymorphisms increases the risk of AD (Kwiatkowski et al., 2016).

Currently, with the advances in molecular biology and genetics, the association between RP and individual gene variations caused by SNPs has become a research focus. Several SNPs have been investigated as the predictors of RP (e.g. *ATM* rs189037 G>A, rs228590 C>T and rs373759 G>A (Xiong et al., 2013); *Lin28B* rs314280 G>A and rs314276 C>A (Wen et al., 2014); *TGF*‐*β1* rs1982073 T>C and rs11466345 A>G (Niu et al., 2012; Yuan et al., 2009); *XRCC1* rs25487 A>G (Yin et al., 2011)). Our previous study found that compared with the *NEIL1* rs4462560 GG, rs4462560 GC/CC variant significantly reduced the occurrence of grade ≥2 acute radiation injury after radiotherapy for esophageal cancer. Although in the past years, several genes have been found that their SNPs were associated with risk of RP in patients with thoracic tumor, they were merely relationship on phenotype. This research deeply explained functional association between *NEIL1* SNP and risk of RP. Furthermore, our study expanded the tumor species and reached the same conclusion in lung cancer, suggesting that *NEIL1* and its genetic polymorphisms play an important role in predicting tumor radiation damage. *NEIL1* SNPs can be regard as molecular markers to predict the occurrence of radiation injury, it also proves evidence about the relationship between genetic variations of core gene in DNA damage repair pathways and the radiation induced inflammation or fibrosis, the mechanism may promote the development of targeted therapy of radiation oncology and individualized treatment, so as to improve the cure rate and limit radiation toxicity simultaneously.

We expect that this result can be used to predicting the occurrence of radiation lung injury in the clinical. For patients carried rs4462560G, the dose of radiotherapy will be adjusted and the monitoring after radiotherapy will be strengthened. The sample size of this study is limited because of single‐center study, we hope to expand the sample size in the future to obtain more reliable results. Besides, several limitations in this study should be mentioned. Firstly, our previously study did not find the significant associations between the risk of RP and rs7402844, its contrary with our current finding. The reasons may as follow: on the one hand, our sample size was limited, further studies with more sample size need to be confirm the relationship between this SNP and RP risk. On the other hand, treatment between patients were different, patients underwent radiotherapy with or without chemotherapy may affect the lung cancer patients. Secondly, as an exploratory study with a limited study power, we didn't explore the mechanism deeply, further exploration of treatment strategies is needed. Thirdly, the predictive value of a single predictor for RP is limited, and the sensitivity and specificity are unsatisfactory. Maybe we are supposed to consider combining multiple SNPs or combining SNP and serum biomarkers to predict the risk of RP. Therefore, more studies support the combination of multiple positive predictors to improve the predictive level of RP.

In summary, our study illustrates the effect of the *NEIL1* gene SNPs on RP risk, that rs4462560 G>C and rs7402844 C>G is significantly associated with RP susceptibility in lung cancer patients after radiotherapy. Future expanded researches will be focused on discovering the mechanism of genetic variations in RP.

## CONFLICT OF INTEREST

The authors declare that they have no conflicts of interest.

## AUTHOR CONTRIBUTIONS

MZ conceived and designed the study. XC provided clinical samples. LZ provided technical support. YZ, JY collected clinical parameters and dosimetric parameters. YZ conducted the experiment. MJ, SZ analyzed data and prepared all the tables. YZ and MZ wrote the manuscript. All authors approved the final manuscript submitted for publication.

## Data Availability

All data are available upon request.
